# Imaging Defective Electronic States in Ultrathin CeO_2_ Nanostructures Grown on Graphene by Pulsed Laser Deposition

**DOI:** 10.1021/acsomega.5c08701

**Published:** 2025-11-04

**Authors:** Diego E. L. Silva, Barbara P. Gonçalves, Nicolas P. Vasconcelos, Rafael R. Barreto, Renato Veloso, Larissa Otubo, Fabio C. Fonseca, Rodrigo G. Lacerda, Angelo Malachias, Rogerio Magalhaes-Paniago, Andre S. Ferlauto

**Affiliations:** † Laboratory of Materials for Energy, Center for Engineering, Modeling and Applied Social Sciences, 74362 Federal University of ABC, Santo André 09210-170, São Paulo, Brazil; ‡ Institute for Energy and Nuclear Research, 119500 IPEN-CNEN, São Paulo 05508-000, Brazil; § Physics Department, Federal University of Minas Gerais, BeloHorizonte 31270-901, Brazil

## Abstract

We report here the growth of ultrathin films of ceria by pulsed laser deposition on HOPG/graphene substrates. The controlled growth of CeO_2_(111) nanoislands on graphene via pulsed laser deposition (PLD) demonstrates a strong dependence on the substrate defect density, where defects serve as preferential nucleation sites. Higher oxygen partial pressure during deposition enhances surface diffusion, promoting the formation of triangular dendritic nanostructures. Scanning tunneling spectroscopy (STS) reveals mutual electronic interactions between the ceria nanoislands and the graphene substrate, while high-resolution STM imaging identifies ordered oxygen vacancy arrays within the CeO_2_ surface. Bias-dependent STM mapping further highlights the complex electronic configuration of the islands. The presence of these ordered defects suggests the potential for precise spatial control, enabling tailored electronic properties through doping or optimized graphene interactions. These findings advance defect-engineered oxide nanostructures, offering promising applications in catalysis, sensing, and optoelectronics via vacancy manipulation in ultrathin films.

## Graphical abstract

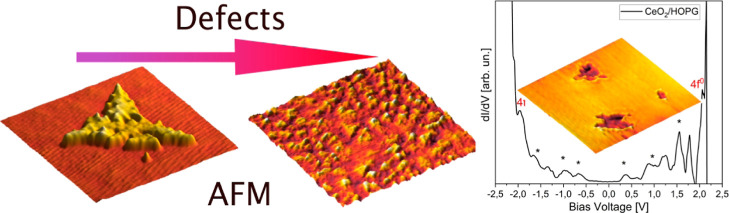

## Introduction

Cerium oxide, CeO_2_, has been widely used in various applications, such as catalysis, fuel cells, sensors, and biomedicine. It is a key component in catalyst converters used in internal combustion engines for removing toxic or dangerous gases (NO_*x*_, CO, and hydrocarbons) due to its unique redox properties, i.e., the capability to easily store and release oxygen, allied with features such as low cost and thermal stability. The good redox properties have also stimulated the study of ceria nanoparticles and thin films as sensors for combustible or toxic gases. Creation or annihilation of oxygen vacancies, depending on the environment, results in changes in electrical conductivity that can be used for sensing.^1–3^ In ceria nanoparticles, the kinetics of oxygen release and capture depend on the crystalline orientation of the exposed facets, and thus, many works have explored the synthesis of ceria NPs with different shapes, such as nanorods, nanocubes, and mixed facet nanoparticles, in attempts to optimize their catalytic and sensing capabilities.^4^ The use of dopants or alloying has also been a widely applied strategy to impart novel properties to ceria. Aliovalent doping with lanthanides such as gadolinium or samarium (in the 10–20 mol % range) results in orders of magnitude increases in the ionic conductivity due to the creation of oxygen vacancies, which makes it suitable for applications in solid oxide fuel cells and membrane reactors.^5^ Additionally, the dopants may act as redox species, enhancing catalytic activity,^6^ or serve as suitable catalytic sites in complex oxide catalyst systems.^7^

The combination of ceria nanostructures with other materials, such as metallic nanoparticles or carbon nanomaterials, can yield strong synergic effects. For example, ceria has been widely used as the support of metal nanoparticles for high-temperature heterogeneous catalysts. Aside from the standard support role, ceria also provides active oxygen species for the desired reactions.^8,9^ On the other hand, graphene has been successfully employed as a support to anchor ceria nanoparticles, helping to stabilize their morphology, tune their redox properties, and enhance the electrical conductivity.^10^

These various studies have highlighted the importance of ceria morphology and faceting as well as its interfacial interactions with substrates and supports. Despite the promising results regarding catalytic and sensing improvements in the CeO_2_ nanocarbon system,^11,12^ there is a lack of studies aiming at the understanding of the morphology of two-dimensional ceria nanostructures and their electronic properties, exploring alternatives for property enhancement and controlled synthesis. In this context, we present here a detailed study of the growth parameters of ultrathin ceria nanostructures on graphene. Using atomic force (AFM) and scanning tunneling (STM) microscopies, the shape, crystallographic orientation, oxygen vacancy concentration, and the impact of such variables on the electronic properties of this material were studied. A detailed scanning tunneling microscopy/spectroscopy (STM/STS) study of the CeO_2_ surface revealed the spatial distribution of in-gap electronic states as a function of energy. The experimental data sheds light into electronic states that can improve CeO_2_/graphene usage on applications such as sensing devices.^13,14^

## Experimental Details

CeO_2_ nanoislands were prepared by pulsed laser deposition (PLD) using a homemade target prepared from commercial CeO_2_ powder. Two PLD parameters were investigated: the UV laser fluence and the oxygen pressure in the PLD chamber. The substrate temperature was kept fixed at 500 °C to minimize changes in the crystalline quality of the graphene substrates due to thermal oxidation. The distance between the target and substrate was also kept constant. The morphology of the produced nanoislands was studied using AFM and STM at ambient temperature and pressure. AFM images were acquired in PeakForce Tapping mode using a Multimode 8 equipped with ScanAsyst-Air probe (resonance frequency ∼70 kHz; spring constant ∼0.4 N/m), both from Bruker.

Three types of substrates were used: mechanically exfoliated multilayer graphene, single-layer graphene produced by chemical vapor deposition (CVD), and highly oriented pyrolytic graphite (HOPG). For mechanical exfoliation, HOPG was used as flake source and SiO_2_ as substrate. For graphene growth via CVD, a tubular furnace connected to a controlled hydrocarbon gas delivery system served as the reactor, with a standard copper (Cu) substrate acting as the catalyst. Prior to growth, a wet transfer process onto the Si/SiO_2_ substrate was performed.

## Results and Discussion

### Growth Study

Initially, the effects of pulsed laser deposition (PLD) parameters on the growth of ceria on exfoliated graphene were investigated. The effect of laser fluence in CeO_2_ deposition is presented in Figure 1 (a–c). The deposition time for all samples was kept the same, and therefore, the laser fluence can be associated with the amount of deposited material. At low fluences, the preferential growth sites for CeO_2_ are located at the crystalline defects on the graphene lattice. Extended defects and surface steps of the multilayer exfoliated graphene provide favorable sites for the initial growth of ceria nanoislands, which are driven by adatom diffusion and surface energy minimization (Figure 1c). Once the edges and the steps between different graphene layers are occupied, the nucleation starts to occur over the flat crystalline substrate surface. The resulting CeO_2_ nanostructures exhibit a triangular dendritic morphology that can be clearly distinguished from the elongated step-grown structures.

**Figure 1:**
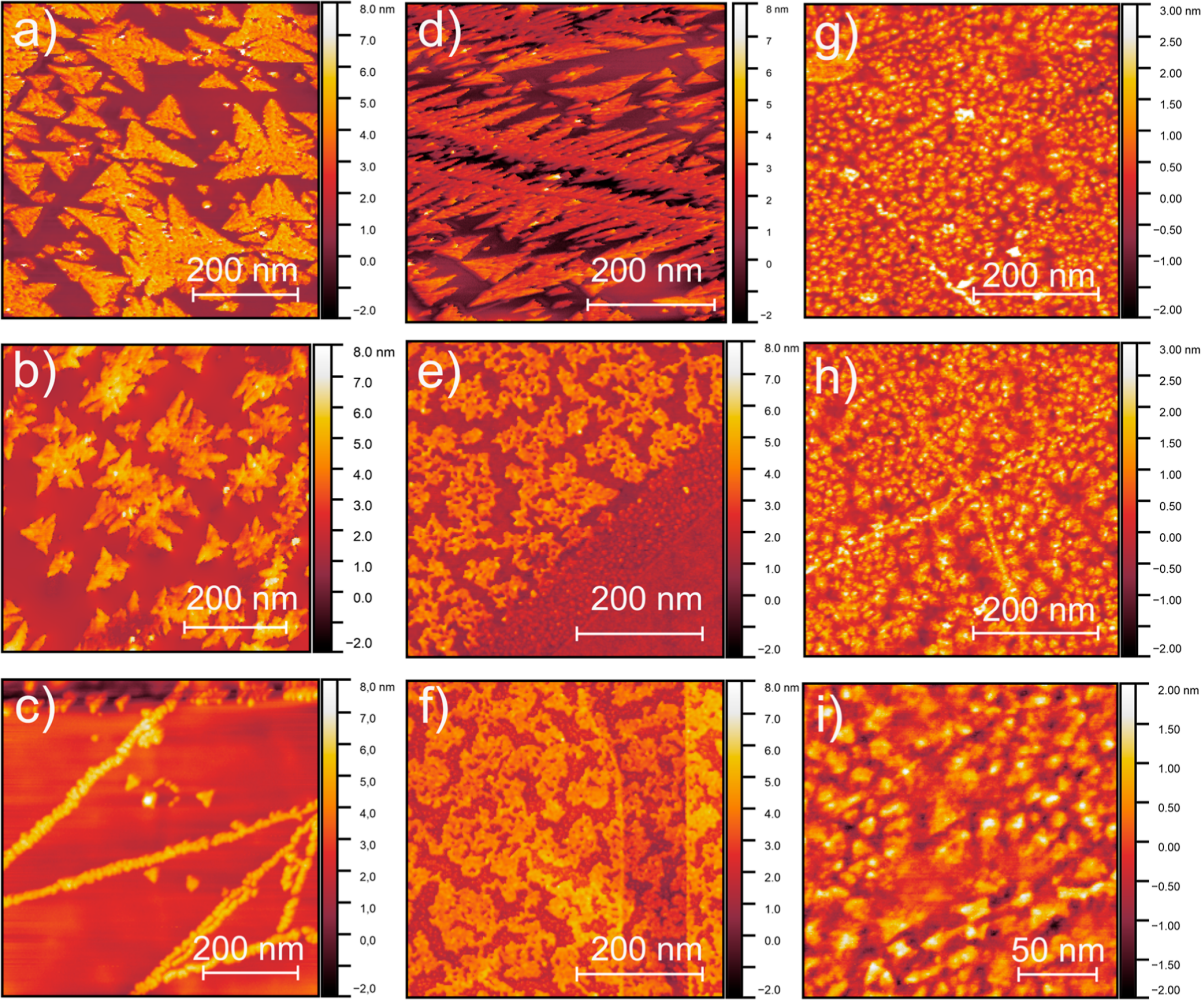
Atomic Force Microscopy. Images of CeO_2_ nanostructures grown on graphene substrates at (a) 75 mJ, (b) 55 mJ, (c) 40 mJ (varying fluence sample series, with fixed pressure of 1.0 × 10^–1^ mbar), (d) 1.0 × 10^–1^ mbar, (e) 1.0 × 10^–2^ mbar, and (f) 1.0 × 10^–3^ mbar (varying pressure sample series, with fixed fluence of 55 mJ). (g, h, and i) Images are from CeO_2_ nanostructures grown on CVD-produced graphene (see text for details).

Triangular-shaped nanostructures have been previously reported in several works and are related to growth with a preferential crystalline orientation. For ceria, the triangular shape is associated with growth along the [111] direction of the fluorite cubic structure that has 3-fold symmetry and has been identified as the most stable orientation.^15,16^ In general, the observed triangular nanostructures present sharper edges when compared to those obtained in this work.^17^ We ascribe this feature to a reduced surface adatom migration due to the relatively low deposition temperature used, when compared to those used in previous works on CeO_2_ ultrathin films. Interestingly, the height of the CeO_2_ nanostructures is similar for all graphene surfaces at ∼2.6 nm, which corresponds to approximately 8 monolayers of 0.31 nm along the [111] direction. Small, coalesced round grains are observed on the dendrite morphology, suggesting that at early growth stages, adatoms are trapped by local substrate defects before coalescing in a cluster. As mechanical exfoliation can produce samples with several layers on each flake, we were able to confirm that there is no evidence of the influence of the number of stacked graphene layers on the shape of the CeO_2_ nanostructures. Indeed, for a HOPG crystal used as a reference substrate, the morphology of the ultrathin CeO_2_ nanostructures is the same as that observed for the nanostructures deposited over the exfoliated multilayer graphene by the same conditions of deposition. Interestingly, graphene is a preferential surface for the ultrathin structure growth; by comparing several AFM measurements, no indication of deposition outside the graphene nanostructures was observed, even for longer deposition times.

Another important parameter in the PLD of oxides is the oxygen partial pressure. To investigate its effect, a series of ultrathin CeO_2_ nanostructures were deposited under different O_2_ partial pressures. It was found that the use of relatively large oxygen partial pressure of oxygen (pO_2_ = 1.0 × 10^–1^ mbar, Figure 1d) produces triangular dendritic nanostructures, whereas the use of lower pO_2_ pressure results in islands having irregular undefined symmetry (Figure 1e,f). This trend suggests that reduced pO_2_ restricts the surface mobility of the growing species, consistent with prior studies. For example, in a study of ceria growth on Cu(111) by Ce evaporation, Höcker et al. observed that higher pO_2_ enhances Ce atoms surface diffusion, leading to square (100)-oriented CeO_2_ islands.^18^ In contrast, the dendritic morphology observed in the present study closely resembles the one observed in another study using Ce evaporation under controlled pO2.^19^ In that case, dendritic CeO_2_ islands are formed on the top of a well-defined triangular 2D CeO_2_(111) interfacial layer formed on the Cu(111) substrate. The dendritic morphology was attributed to a low surface diffusion of cerium on the interlayer ceria. In summary, even though multiple factors shape the ceria island morphology, our results confirm that the growing species surface diffusion is enhanced with increasing pO_2_.

To study the differences in growth due to the properties of the graphene substrate, a single-layer graphene sample grown by CVD was also used as a substrate for CeO_2_ PLD deposition. The PLD deposition parameters were similar to the previous samples: fluence of 55 mJ and an O_2_ pressure of 1.0 × 10^–1^ mbar. AFM images show differences between growth on distinct graphene types (exfoliated and CVD) (Figure 1g–i). While in exfoliated samples, ultrathin CeO_2_ growth leads to triangular-shaped nanoislands, the use of CVD graphene as the substrate results in the formation of smaller clusters with undefined shape and average height of ∼0.8 nm. In addition, enhanced step-edge line clustering is observed along the whole sample that can be associated with extended defects.

The lack of well-defined ceria nanoisland formation on the graphene CVD samples can be attributed to the large concentration of defects that arises from two sources: usually, CVD graphene has a lower crystallinity (smaller domain size) and higher concentration of point defects as compared to mechanically exfoliated graphene. In addition, the wet chemical process used to remove CVD graphene from Cu also can leave residues on its surface. A closer look to AFM images carried out in smaller areas allow the observation of some features formed around the deposited CeO_2_ on the graphene surface. We observed that the nanoislands are formed by the coalescence of small clusters into these larger structures (Figure 2). Upon careful analysis, these small clusters are found at the edges between graphene layers and on the CVD graphene itself. This suggests that interaction with these defective or incomplete sp^2^-terminated edges inhibits the formation of dendritic nanoislands.

**Figure 2:**
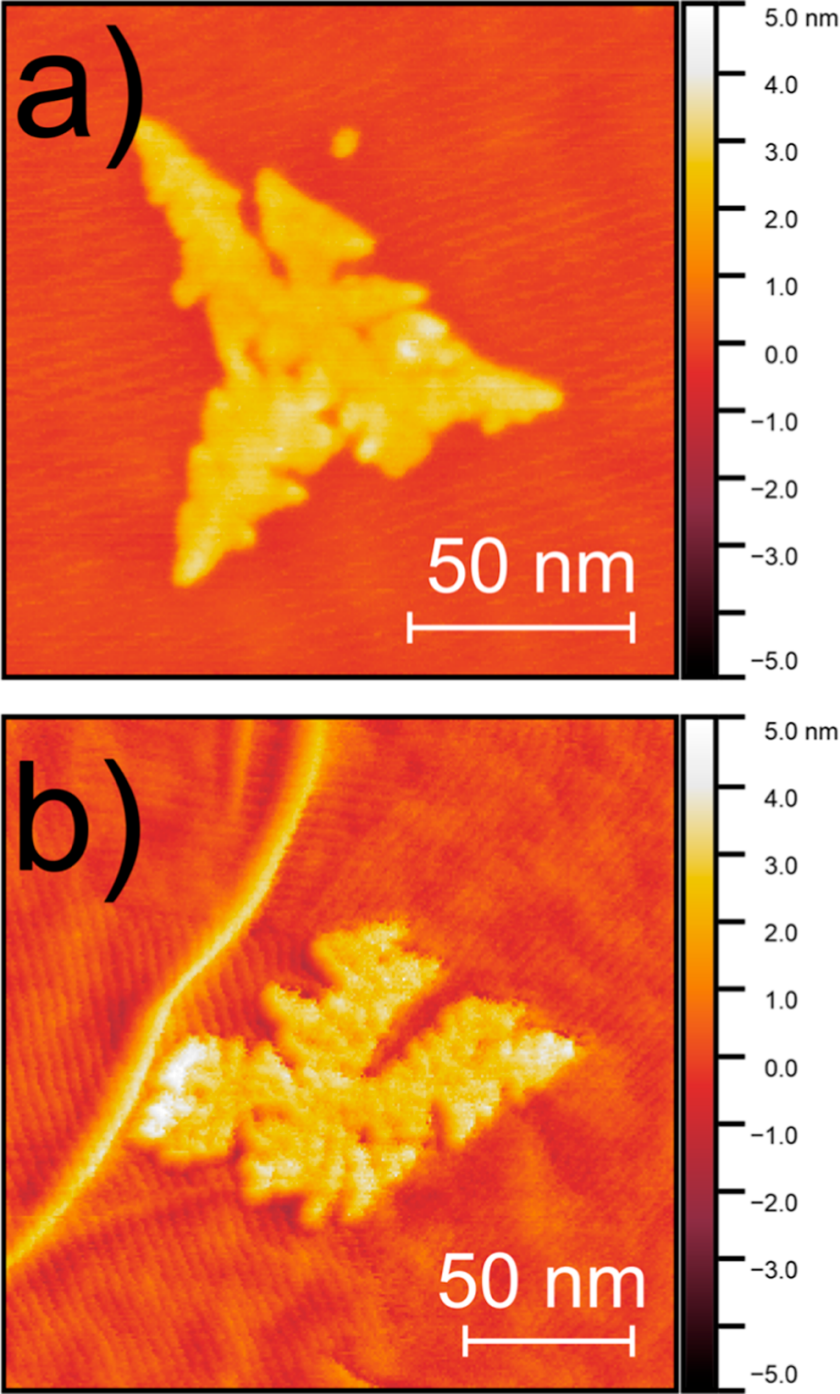
AFM images showing the perturbations caused on the carbon structure by the CeO_2_ deposition. (a) Graphene substrate and (b)­HOPG substrate.

The results of Figures 1 and 2 indicate that in applications where high surface area ceria clusters are desirable to provide higher interaction with the gaseous or liquid environment, such as sensing or catalysts, the use of CVD graphene can be desirable.

From AFM images (Figure 2), it was possible to observe some features formed around the deposited CeO_2_ on the graphene surface; this could be caused by the interaction and the mismatch between the atomic structure of the sp^2^ carbon and the oxide. As the wrinkles could promote strain on the atomic structure of the carbon material, we measured STS at different points in the proximity of the ceria flakes as presented in the next section.

### Spectroscopic Study

A NaioSTM Scanning Tunneling Microscope from NanoSurf was used to measure the CeO_2_/HOPG heterostructures at ambient pressure and room temperature. Figure 3a shows an STM image depicting two CeO_2_ nanostructures grown on HOPG using the same PLD conditions as those of the structures shown in Figure 1b. Due to the nature of STM measurements, the CeO_2_ nanostructures appear as depressions rather than elevations, as observed in AFM, because of the higher electronic conductivity of HOPG relative to CeO_2_. In constant-current STM imaging with a fixed bias and set point current, the tip moves closer to the surface when scanning over the less conductive CeO_2_, resulting in an apparent topographic depression.

**Figure 3:**
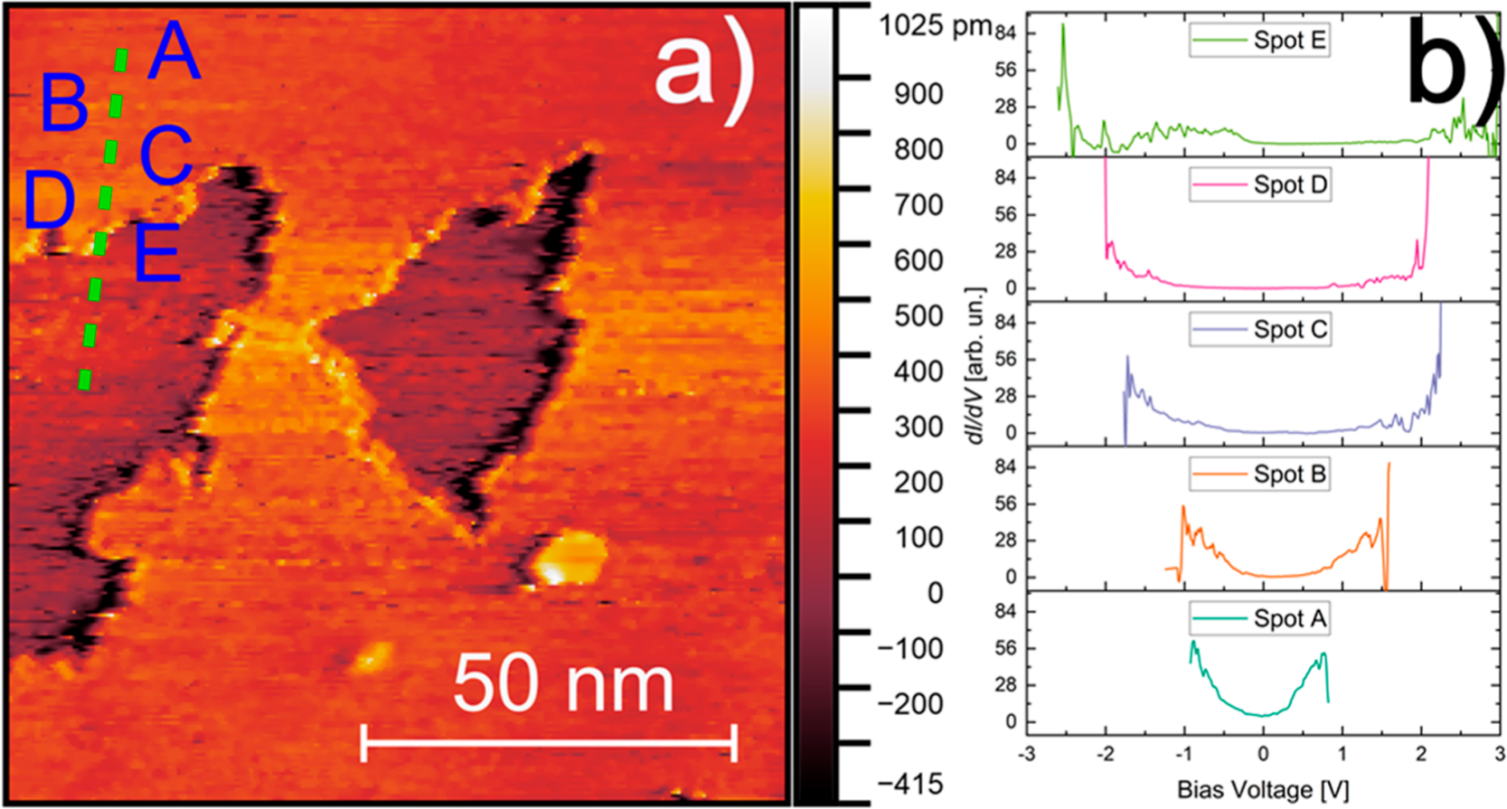
(a) STM image of the nanostructures o the oxide. The green spots represent the places were the STS data were collected. (b) Differential conductance spectra taken on the spots marked in a.

Figure 3 shows the scanning tunneling spectra (STS) collected at different points in the proximity of the ceria flakes. It reveals the effect of the interaction between graphene and the CeO_2_ overlayer. As graphene step edges and wrinkles due to the formation of the CeO_2_ island (Figure 2), the result strain is reflected in its electronic states. The differential conductance (*d*I/*d*V) spectra depicted here exhibit a clear bandgap when measured atop the ceria surface (Figure 3, spot E), changing to a narrow gap and a metallic behavior (V-shaped) as one moves the tip into the HOPG surface. Even though STM/STS measurements were performed at room temperature and ambient pressure, it was possible to clearly observe a strong electronic interaction between the CeO_2_ nanostructures and the underlying graphitic layer. A slight shift of the Fermi level (zero bias) to higher voltage values was also observed, indicating a p-type doping behavior induced by the substrate presence, confirming the change in the electronic structure of the HOPG due to the presence of the CeO_2_ overlayer.

An atomically resolved STM image collected over a ceria flake is shown in Figure 4. It reveals a hexagonal lattice pattern, typical of a (111) CeO_2_ surface. Moreover, previous works have shown that oxygen vacancies appear as brighter spots on STM of CeO_2_ surfaces.^20,21^ A careful analysis of Figure 4 suggests that ordered arrays of oxygen vacancies are present in the imaged ceria nanoisland, as indicated by the triangles drawn as a guide to the eyes. Even though the ordering does not extend along the entire mapped surface, such local arrangements could be enough to affect the electronic structure of the larger areas.

**Figure 4:**
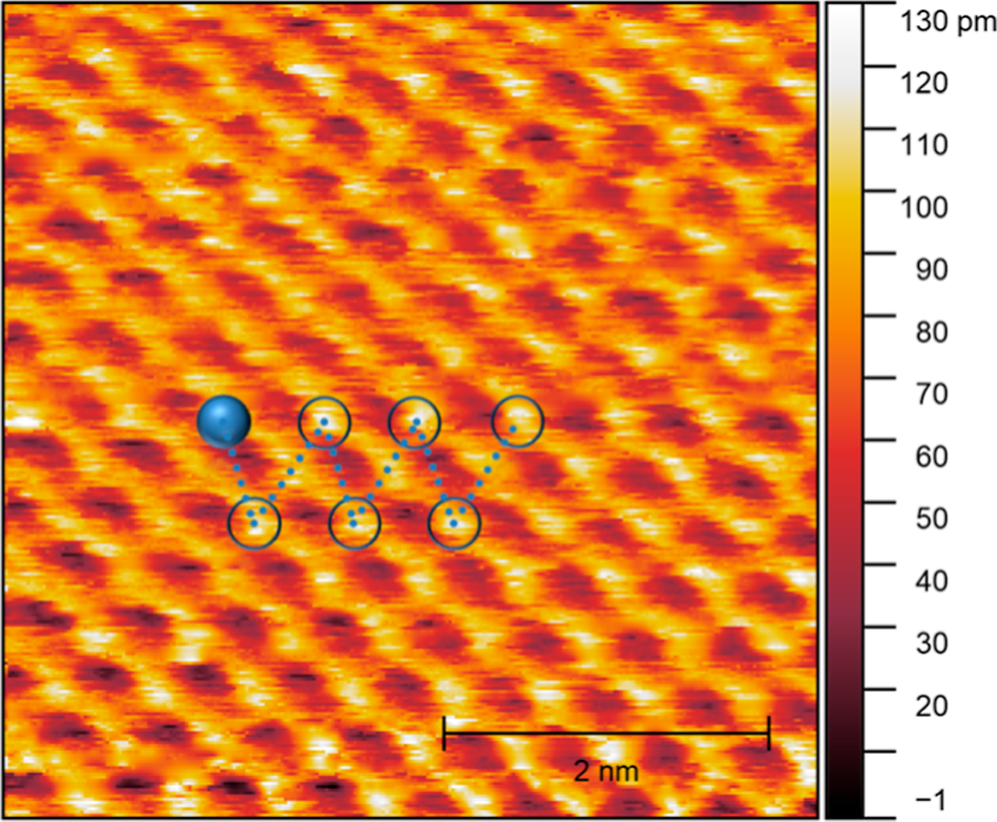
Atomic resolution STM image of Ceria/HOPG. The dashed triangles show the ordering of the oxygen vacancies on the oxide structure.

For ultrathin CeO_2_ nanostructures, Radovic et al.^22^ observed variations on the dI/dV spectra of ceria nanoparticles depending on the synthesis method. They measured the effects of filling of 4f states but also measured additional states within the bandgap related to color centers. These defects have been previously reported for CeO_2_ nanostructures.^23,24^ Hence, for the ceria nanoislands studied in this work, 2D confinement effects and/or interaction with the HOPG substrate should result in more complex spectra as compared to ceria NPs. For example, oxygen vacancies, F centers, and polarons can interact and group in specific regions of ceria nanoislands. By varying the applied voltage, it is possible to map different electronic states across the nanoislands. This is evident in the STM images presented in Figure 5, in which the observed topography of the islands changes with applied bias. Different plateaus are observed in the nanoislands at different positions, indicating the presence of regions having different electronic structures within the same island. In addition, brighter borders are observed for *V* = −0.5 V, suggesting the presence of edge-induced one-dimensional confined electronic states. These features can be related with the presence of localized defects and grain boundaries within the nanoislands. The presence of oxygen vacancies, for instance, can generate perturbations in the crystalline structure of ceria, allowing the formation of polarons and F centers or even serving as precursors for local phase transitions.^25–30^ Moreover, the interaction of the ceria island with the underlying carbon structure may disturb its electronic structure, providing additional electrons that interact with the naturally formed vacancies, giving rise to the variations observed using different tip bias.

**Figure 5:**
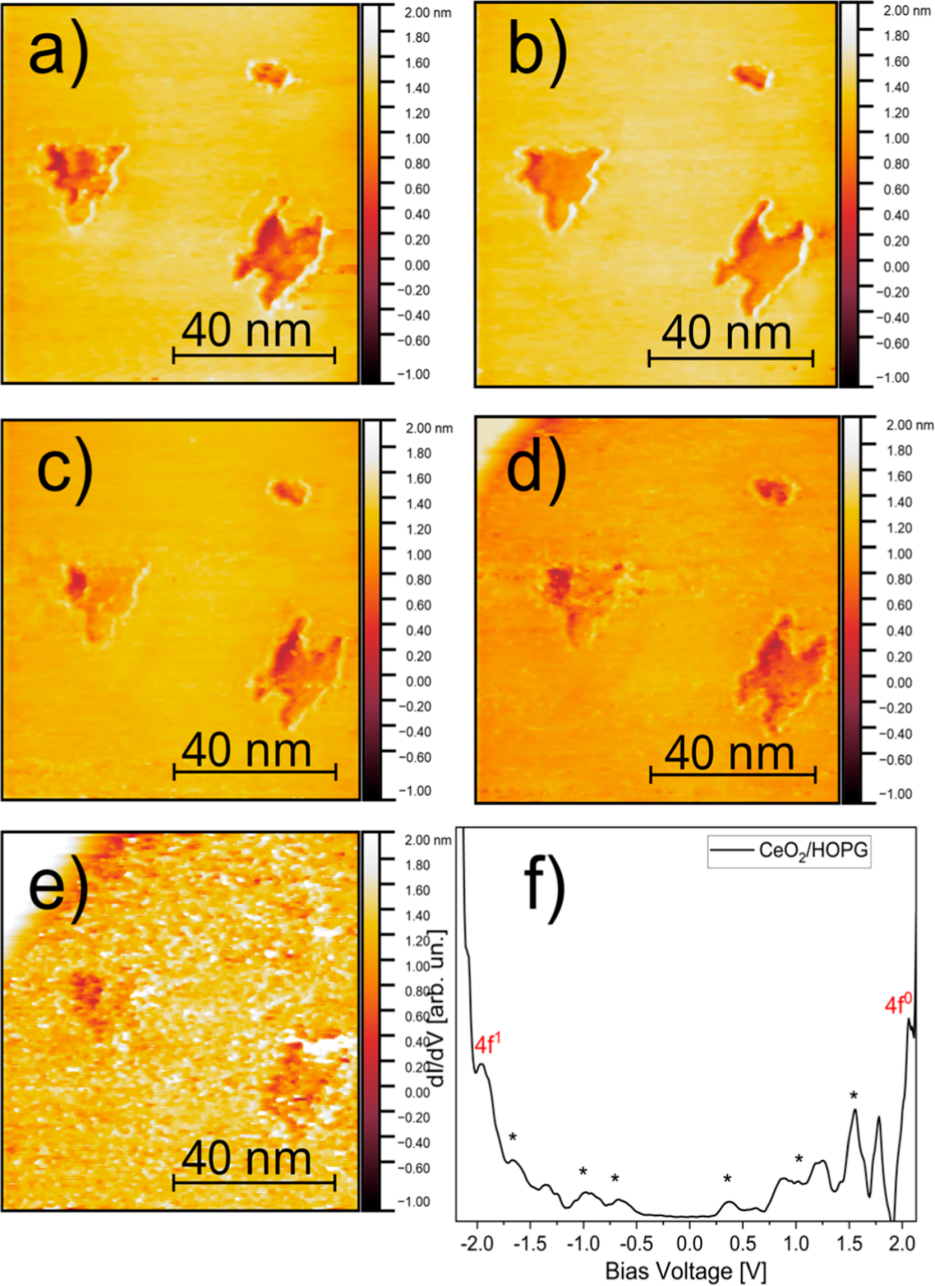
STM images measured at different voltages: (a) −0.5 V, (b) −1 V, (c) −1.5 V, (d) −2 V, and (e) −2.5 V. (f) Characteristic differential conductance spectra of CeO_2_ nanostructures. The asterisk shows confined states formed by defects.

A complete STS spectrum collect over a ceria nanoisland is shown in Figure 5f, where defect electronic states corresponding to some of the bias-selected images of Figure 5a–e were carried out. Such a spectrum, as well as the possibility of imaging CeO_2_ nanostructures at tip bias within the material bandgap, evidence the large contribution of defects to the local density of states.

## Conclusion

Controlled growth of CeO_2_(111) nanoislands on graphene via pulsed laser deposition (PLD) reveals a strong correlation between substrate defect density and nanostructure morphology, with substrate defects acting as preferential nucleation sites. In addition, relatively higher oxygen partial pressure during deposition induces enhanced surface diffusion of growing species favoring the formation of triangular dendritic shapes.

Differential conductance spectroscopy (STS) reveals a strong influence of the ceria nanoisland on the electronic structure of the graphene substrate and conversely the effect of the sp2 carbon structure on the electronic structure of the CeO_2_ islands. STM high-resolution images indicate the presence of arrays of oxygen vacancies within the surface of CeO_2_ islands, whereas STM mapping using varying bias shows that the islands have a rich and complex electronic configuration. The presence of ordered defects indicates potential for precise spatial defect control that could pave the way for tailoring CeO_2_ electronic properties, achievable through targeted doping or optimized graphene interactions. These findings advance the development of defect-engineered materials, enabling applications in catalysis, sensing, and optoelectronics via vacancy manipulation in ultrathin oxides.

## Notes

Diego E. L. Silva; Center for Natural and Human Sciences; Federal University of-ABC,09210-170, Santo Andre, São Paulo, Brazil
